# Twelve tips for introducing the concept of validity argument in assessment to novice medical teachers in a workshop

**DOI:** 10.15694/mep.2021.000074.1

**Published:** 2021-03-25

**Authors:** Hosam Eldeen Gasmalla, Majed Wadi, Mohamed H. Taha

**Affiliations:** 1 Al-Neelain University; 2 Medical Education Department; 3 University of Sharjah

**Keywords:** Validity evidence, Assessment, Measurement

## Abstract

This article was migrated. The article was not marked as recommended.

**Background:** Misconceptions have been observed in the reporting of validity in a significant amount of the published work in the field of students’ assessment as well as in its application by faculty; this calls for action concerning the dissemination of information about the concept of validity in relation to assessments, especially among novice medical teachers.

**Aim:** This work aims to guide how the concept of validity argument in assessment is delivered to novice medical teachers in a workshop.

**Methods:** Critical reflection and a careful review of relevant literature were used to develop these tips.

**Results and Conclusion:** Twelve tips are introduced to support the ability of instructors that are conducting a workshop to introduce the concept of validity, especially to novice medical teacher.

## Introduction

The interpretation of the term validity has changed over time. In the 1920s and up to the 1950s, it referred to the degree to which a test measures what it is supposed to measure. During that timeframe, emphasis was placed on the idea of validity being the character of the test; at that time, criterion validity and content validity were two known types of validity, the criterion validity has been defined as the validity of the test in which the correlation of test scores to a given criterion is determined ( Colliver, Conlee and Verhulst, 2012), while the content validity referred to the relation between the content of the assessment tool and the measured construct ( Artino *et al.,* 2014). Thus, tests were defined as being either valid or not. In the mid-1950s, a third type of validity emerged: construct validity. This was due to the struggle to define the criterion/reference standard of some traits, such as clinical reasoning, through criterion and content validity because, by that time, the concept of validity was understood as meaning the validity of the test for an explicit purpose. In the 1980s, the contemporary notion of validity evolved, and it was seen as a unitary concept that evolves around the construct ( AERA, APA and NCME, 1999). The concept of validity has continued to evolve; it is now focused on the suitability and appropriateness of the interpretations made from the assessment scores ( Oermann and Gaberson, 2019).

Misconceptions and malpractices have been noted in the reporting of validity in the published work that focuses on different types of assessment methods ( Gasmalla and Tahir, 2020). The practice of validating the interpretation of test scores has been described as being below than optimal ( St-Onge and Young, 2015). Investigators often apply deficient approaches to the validity argument ( Cook *et al.,* 2015). Furthermore, there is uncertainty among researchers regarding the type and amount of validity evidence that must be collected and presented to sufficiently support the inferences, and the identification of the applied validity framework is either incomplete or absent; sometimes, outdated frameworks are used ( Cook *et al.,* 2014). With various conceptualisations of validity, researchers do not adhere to a unified approach in reporting the validity of an assessment ( Young *et al.,* 2018).

Since misconceptions have been observed in how the concept of validity is understood, action is needed to clarify how to disseminate information about this concept. This work aims to provide guidance about delivering the concept of validity argument in assessment to novice medical teachers in a workshop. If the faculty development unit or an expert chooses a workshop as a training method, this work is especially suited for that format. It is based on the author’s experience and that of others cited in the literature.

## Tip 1. Consider the number of participants

It is imperative to limit the number of workshop participants to a range of 5 to 15. This will allow for better engagement in the discussion, and it will allow the participants to benefit from the exercises offered during the workshop ( McLeod *et al.,* 2010). After determining the number of participants, it is crucial to conduct the Training Needs Assessment (TNA). TNA helps identify the faculty’s current level of knowledge about the concept of validity, and it compares that competency level of knowledge to the required standard. Hence, it is used to determine training needs rather than simply assuming that all faculty need training or the same level of training about the concept of validity. This is further explored in the next tip.

## Tip 2. Explore the workshop participants’ prior knowledge

There are many validity frameworks. Traditionally, validity is classified as content, criterion and construct validity ( Goodwin and Leech, 2003), validity is also viewed as being a unitary concept ( Downing, 2003), and there is Kane’s framework for validation ( Cook *et al.,* 2015). Since novice medical teachers may have varying degrees of prior knowledge about the concepts of validity, it is crucial to check the faculty’s understanding of validity before starting the training.

The foundational base of exploration of prior knowledge is based on the constructivism learning theory. That theory posits that new knowledge and comprehension develop by building on an individual’s existing understanding ( Badyal and Singh, 2017). Learners construct knowledge based on their experiences, and they assimilate, accommodate, and adapt knowledge to develop a new understanding ( Torre *et al.,* 2006). The learning process entails the role of critical reflection, which is based on prior experience, to construct meaning ( Cirocki and Farrell, 2017). The different validity frameworks could be contributing to the confusion about validity, especially with the ongoing discussion that challenges the concept of construct validity ( Colliver, Conlee and Verhulst, 2012). Thus, it is useful to start by exploring what the participants know about validity. Prior knowledge is explored to enrich the discussion; the subsequent debate and disagreement among the workshop participants will help ensure that their minds are open and receptive to what is coming next.

## Tip 3. Consider the historical evolution of the concept

After exploring the different views about validity, it is time to provide an explanation of it by discussing the historical evolution of the concept. This will help later when delivering the concept of validity. For simplification, this information can be presented in the chronological order of eras of the evolution of the concept; for example: the era of content and criterion validity (from the 1920s and to the 1950s), the introduction of the construct validity in the mid-1950s ( Cronbach and Meehl, 1955), which led to the era of the tripartite concept, and finally the introduction of the unitary concept of validity ( AERA, APA and NCME, 1999). During this overview, it is imperative to explain why the tripartite concept has failed to grasp the full magnitude of validity, and that the definition of validity has evolved from focusing on the validity of the test to the validity of its use for a particular purpose, and eventually, the validity of the interpretations drawn from the test scores. This will smoothly lead the participants into the concept of a *construct* since all the aspects of validity are centred around it.

## Tip 4. Explain the concept of a construct

Since the contemporary concept of validity has been seen as a unitary concept that evolves around the construct, it is important to discuss what is meant by a construct. A construct indicates a characteristic that a test aims to measure ( AERA, APA and NCME, 1999). In this step, the workshop participants are asked to take their time explaining this concept, as later it will help them comprehend the importance of defining the construct and linking all the other validation activities to it. Thus, the purpose of this step is to explain how other types of validity contribute in one way or another to construct validity.

## Tip 5. Start backwards: Discuss the threats to validity

At this point in the workshop, it is not yet time to introduce the five sources of validity evidence. From our experience, it is useful to present a scenario: you are in a faculty board meeting or department meeting, and one of your colleagues has presented a test score with a massive failure. In your opinion, what could have possibly gone wrong? This question will generate a discussion about threats to validity. You will not have to mention them; you will only need to categorise the examples the participants provide into construct-overrepresentation and construct-irrelevant variance ( Downing and Haladyna, 2004). Thus, the purpose of this step is to introduce the workshop participants to the threats to validity.

## Tip 6. Ask the workshop participants to define the impact of the threats on the inference

At this point in the workshop, you may lead the discussion into the effects of those threats on validity. The overall process requires the participants to present examples of threats to validity, describe the effect and, finally, suggest the actions that need to be taken to eliminate those threats (see Tip 7).

**Figure 1.**
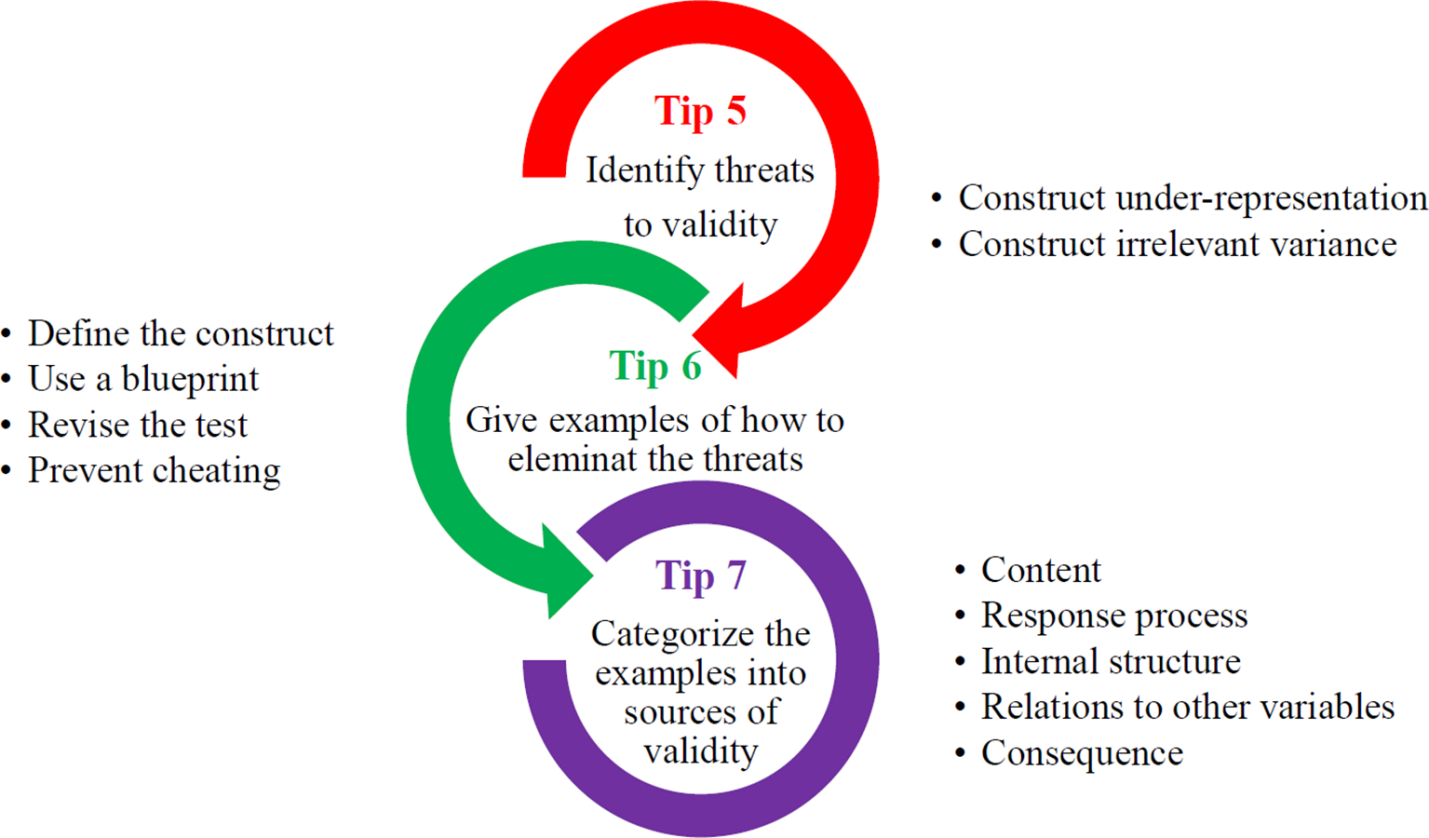
Summary of the process in Tip 5, Tip 6 and Tip 7

This tip (Tip 6) will usually intermingle with the previous tip and the next tips. The following diagram ( Figure 1) explains the process for Tip 5, Tip 6 and Tip 7.

## Tip 7. Ask the participants to provide examples of how to eliminate the threats to validity

Downing has provided a comprehensive guide describing the threats to validity, including examples of how to eliminate those threats ( Downing, 2003). Examples of the action taken to eliminate the threats to validity will automatically drive the participants into thinking about providing evidence for those actions, which will comprise evidence of validity. This will pave the way to introduce the five sources of evidence of validity. It is useful to conduct an exercise in which every possible evidence is categorised into the five sources; this exercise will maximise the participants’ engagement.

## Tip 8. End at the beginning: Explain the unitary concept of validity

Now that the participants are familiar with the history of the changes in the concept of validity, the notion of construct validity, validity threats and the five sources of validity evidence, it is time to tie it all together and solidify the unitary concept of validity. By this point in the workshop, you will find that your job has become easy.

## Tip 9. Ask the participants to compare the tripartite concept of validity to the current concept of validity

Well, do not stop there. By this time, some of the participants will still be stuck in the old concept of validity (that is based on the tripartite notion). Goodwin and Leech provided a useful way to aid in the participants’ transition from the tripartite concept to the unitary concept by comparing the types of validity evidence between the old concepts and the current concepts ( Goodwin and Leech, 2003). The purpose of this step is to ease the transition for the participants that are still trapped in the past.

## Tip 10. Address the misconceptions

It is highly likely that the comparison between the tripartite concept and the current concept will show you how many misconceptions are still out there. Examples of misconceptions include: using the phrase *validity of a test* instead of *validity evidence that supports the interpretation,* or the idea that *any number (or type) of validity evidence is enough to build the case* rather than the importance of each evidence in relation to the construct and the assessment tool. Furthermore, some may think that *validation is just a collection of evidence* rather than a whole process that includes identifying the construct and evaluating the evidence. One of the false impressions is that validity is a dichotomy (it is either valid or not valid) instead of being a degree. Some estimate the reliability and rely on it as the sole source of validity, while others think that *after establishing a validity argument for a test, this test is valid for any further/different uses.*

## Tip 11. Ask the participants to validate the test scores from their own previously developed tests

At this point, it is time for the participants to apply what they have learned. Thus, it is necessary to ask them to bring any previous test they have developed along with the test scores. They can work in groups. Their validation activity might raise some questions that were addressed previously or it might reveal unnoticed misconceptions. As the instructor, you must be patient since the concept of validity is complex, ever-evolving and an area that is undergoing continuous debate.

## Tip 12. Post-credit scene: Promote a culture of quality assurance

By the end of the workshop, everyone asks: why are we bothering ourselves with this complex concept? Well, it is time to promote a culture of quality assurance. Considering that the workshop has gone as planned, the participants will find themselves thinking about quality assurance at every step in their career in education. In fact, the promotion of quality assurance is immersed in every aspect of the workshop, and although the intended purpose was to introduce the concept of validity, quality assurance always comes along.

## Conclusion

This work aims to provide guidance about delivering the concept of validity argument in assessment to novice medical teachers in a workshop. Critical reflection and a careful review of the literature were applied to develop these tips. Thus, 12 tips were introduced to support the ability of instructors to conduct a workshop to deliver the concept of validity, especially among novice medical teachers. The tips focused on the history of the evolution in the concept of validity, the notion of construct validity, validity threats and the five sources of validity evidence; they also addressed the common misconceptions that accompany the application. These tips were arranged in such a way to help the workshop participants navigate the steps needed to better understand the concept of validity.

## Take Home Messages

• The interpretation of the term validity has changed over time.
• Misconceptions and malpractices have been noted in the reporting of validity.
• Introducing the concept of validity requires a systematic approach. This approach includes: considering the historical evolution of the concept, discussing the threats to validity, explaining the unitary concept of validity, comparing the tripartite concept of validity to the current concept of validity, addressing the misconceptions and promoting a culture of quality assurance.
• Conducting training need assessment followed by an effective faculty development program is the backbone of this approach.

## Notes On Contributors

**Hosam Eldeen Elsadig Gasmalla** is an assistant professor of human anatomy and histology in the faculty of Medicine, Alneelain University and a researcher in the Education Development Center, Sudan International University. ORCID ID: https://orcid.org/0000-0003-2590-8587

**Majed Mohamed Saleh Wadi** is a senior lecturer of Medical Education, College of Medicine, University of Qassim, KSA. He is the Head of Medical Education Department. Head of the Assessment Committee. ORCID ID: https://orcid.org/0000-0002-8117-770X

**Mohamed Hassan Taha** is a visiting Assistant Professor of Medical Education at the College of Medicine and Medical Education Centre, University of Sharjah, UAE. He is the chair of the Curriculum Committee as well as the Faculty Development Committee. ORCID ID: https://orcid.org/0000-0003-0808-5590
