# Twelve tips for introducing the concept of validity argument in assessment to novice medical teachers in a workshop

**DOI:** 10.15694/mep.2021.000074.2

**Published:** 2021-09-21

**Authors:** Hosam Eldeen Gasmalla, Majed Wadi, Mohamed H. Taha

**Affiliations:** 1 Al-Neelain University; 2 Al-Neelain University; 3 Medical Education Department; 4 Medical Education Department; 5 University of Sharjah

**Keywords:** Validity evidence, Assessment, Measurement

## Abstract

This article was migrated. The article was marked as recommended.

**Background:** Misconceptions have been observed in the application of validity by faculty and in the reporting of validity in a significant amount of published work in the field of students’ assessment. As a result, actions concerning the dissemination of information about the concept of validity in relation to assessments, especially among novice medical teachers, is needed.

**Aim:** This work aims to guide how the concept of validity argument in assessments is delivered to novice medical teachers in a workshop.

**Methods:** Critical reflection and a careful review of relevant literature were used to develop these tips.

**Results and Conclusion:** Twelve tips were introduced to support instructors conducting workshops on introducing the concept of validity, especially to novice medical teachers.

## Introduction

The interpretation of the term validity has changed over time. In the 1920s and up to the 1950s, it referred to the degree to which a test measures what it is supposed to measure. During that timeframe, emphasis was placed on the idea of validity being the character of the test; at that time, criterion validity and content validity were the two known types of validity. When the correlation of test scores to a given criterion is determined, this is referred to as criterion validity ( Colliver, Conlee and Verhulst, 2012), while content validity refers to the relation between the content of the assessment tool and the measured construct ( Artino *et al.*, 2014). Thus, tests were defined as being either valid or not. In the mid-1950s, a third type of validity emerged: construct validity. This was due to the struggle to define the criterion/reference standard of some traits, such as clinical reasoning, using criterion and content validity because, by that time, the concept of validity was associated with an explicit purpose. In the 1980s, the contemporary notion of validity evolved, and it was seen as a unitary concept evolving around the construct ( AERA, APA and NCME, 1999). The concept of validity has continued to evolve; it is now focused on the suitability and appropriateness of the interpretations made from assessment scores ( Oermann and Gaberson, 2019).

Misconceptions and malpractices have been noted in the reporting of validity in the published work that focuses on different types of assessment methods ( Gasmalla and Tahir, 2020). The practice of validating the interpretation of test scores has been described as being less than optimal ( St-Onge and Young, 2015). Investigators often apply deficient approaches to the validity argument ( Cook *et al.*, 2015). Furthermore, there is uncertainty among researchers regarding the type and amount of validity evidence that must be collected and presented to sufficiently support their inferences, and the identification of the applied validity frameworks is either incomplete or absent. Sometimes, outdated frameworks are used ( Cook *et al.*, 2014). With various conceptualizations of validity, researchers do not adhere to a unified approach when reporting the validity of an assessment ( Young *et al.*, 2018).

Since misconceptions have been observed regarding how the concept of validity is understood, action is needed to clarify the best ways to disseminate information about this concept. This work aims to provide guidance on educating novice medical teachers on the concept of validity with regard to assessments via a workshop. The characteristics of a novice medical teacher have been described by Persky and Robinson (2017), and these include the tendency to abide by rules, guidelines, and plans. They need to obtain information in order to learn, and they spend time on the recall process.

This work is especially suited for faculty development units or experts who choose to conduct workshops as a training method. It is based on the author’s experience and those cited in the literature.

## Tip 1. Consider the number and the needs of participants

It is imperative to limit the number of workshop participants to a range of 5 to 15. This will allow for better engagement in the discussion, and it will allow the participants to benefit from the exercises offered during the workshop ( McLeod *et al.*, 2010). However, restricting the number of participants to 15 may not always be a feasible option. In such cases, the instructor/facilitator of the workshop is advised to pay more attention to strategies that can improve the engagement of the participants, such as adding more interactivity, emphasizing dialogue-based styles, integrating storytelling, and adopting tech-enabled approaches. Previous studies have emphasized the benefits of these approaches ( Kinsella, Mahon and Lillis, 2017). After determining the number of participants, it is crucial to conduct a training needs assessment (TNA). A TNA helps in the assessment of the faculty’s current level of knowledge about the concept of validity, and it compares that level of knowledge to the required standard. Rather than simply assuming that all faculty need training or the same level of training on the concept of validity, organizers should use this tool to determine training needs. This point is further explored in the next tip.

Many factors affect the structure and duration of the workshop, such as the number of participants and the TNA. A suggested timetable is attached in the appendix ( Table 1).

## Tip 2. Explore the workshop participants’ prior knowledge

There are many validity frameworks. Traditionally, validity has been classified into content, criterion, and construct validity ( Goodwin and Leech, 2003). Validity is also viewed as being a unitary concept ( Downing, 2003), and there is Kane’s framework for validation ( Cook *et al.*, 2015). Since novice medical teachers may have varying degrees of prior knowledge about the concept of validity, it is crucial to assess their understanding of validity before starting the training.

The exploration of prior knowledge is based on constructivism learning theory. This theory posits that new knowledge and comprehension develop when an individual’s existing understanding is built upon ( Badyal and Singh, 2017). Learners construct knowledge based on their experiences, and they assimilate, accommodate, and adapt knowledge to develop a new understanding ( Torre *et al.*, 2006). The learning process entails the role of critical reflection, which is based on prior experiences, to construct meaning ( Cirocki and Farrell, 2017). The different validity frameworks could be contributing to the confusion about validity, especially with the ongoing discussion that challenges the concept of construct validity ( Colliver, Conlee and Verhulst, 2012). Thus, it is useful to start by exploring the participants’ knowledge of validity. Prior knowledge is explored to enrich the discussion, and the subsequent debate and disagreement among the workshop participants will help ensure that their minds are open and receptive to what comes next.

## Tip 3. Consider the historical evolution of the concept

After exploring the different views on validity, it is time to provide an explanation of it by discussing the historical evolution of the concept. This will help later during the explanation of the concept of validity. For simplification, this information can be presented in the chronological order of the evolution of the concept. For example, the presentation can begin from the era of content and criterion validity (1920s-1950s), followed by the introduction of construct validity in the mid-1950s ( Cronbach and Meehl, 1955), which led to the era of the tripartite concept, and finally the introduction of the unitary concept of validity ( AERA, APA and NCME, 1999). During this step, it is imperative to explain why the tripartite concept has failed to grasp the full magnitude of validity and that the definition of validity has evolved from a focus on the validity of the test to the validity of its use for a particular purpose and, eventually, the validity of the interpretations drawn from the test scores. This will smoothly guide the participants toward the concept of a *construct* since all the aspects of validity are centered around it.

## Tip 4. Explain the concept of a construct

Since the contemporary concept of validity has been seen as a unitary concept that evolves around the construct, it is important to discuss what is meant by a construct. A construct is a characteristic that a test aims to measure ( AERA, APA and NCME, 1999). It is an intangible learner characteristic that cannot be detected directly. Examples include the ability to solve clinical problems or the ability to recall. In this step, the workshop participants are asked to take their time explaining this concept, as it will later help them comprehend the importance of defining the construct and linking all the other validation activities to it. Thus, the purpose of this step is to explain how other types of validity contribute in one way or another to construct validity.

## Tip 5. Start backwards: Discuss the threats to validity

At this point in the workshop, it is not yet time to introduce the five sources of validity evidence. From our experience, it is useful to present a scenario: you are in a faculty board meeting or department meeting, and one of your colleagues has presented the test score of a student who has failed. In your opinion, what could have possibly gone wrong? This question will generate a discussion about threats to validity. You will not have to mention them; you will only need to categorize the examples the participants provide into construct-overrepresentation and construct-irrelevant variance ( Downing and Haladyna, 2004) Thus, the purpose of this step is to introduce the workshop participants to the threats to validity.

## Tip 6. Ask the workshop participants to define the impact of the threats on inference

At this point in the workshop, you may lead the discussion into the effects of threats on validity. The overall process requires the participants to present examples of threats to validity, describe the effects, and finally, suggest the actions that need to be taken to eliminate those threats (see Tip 7).

**Figure 1.**
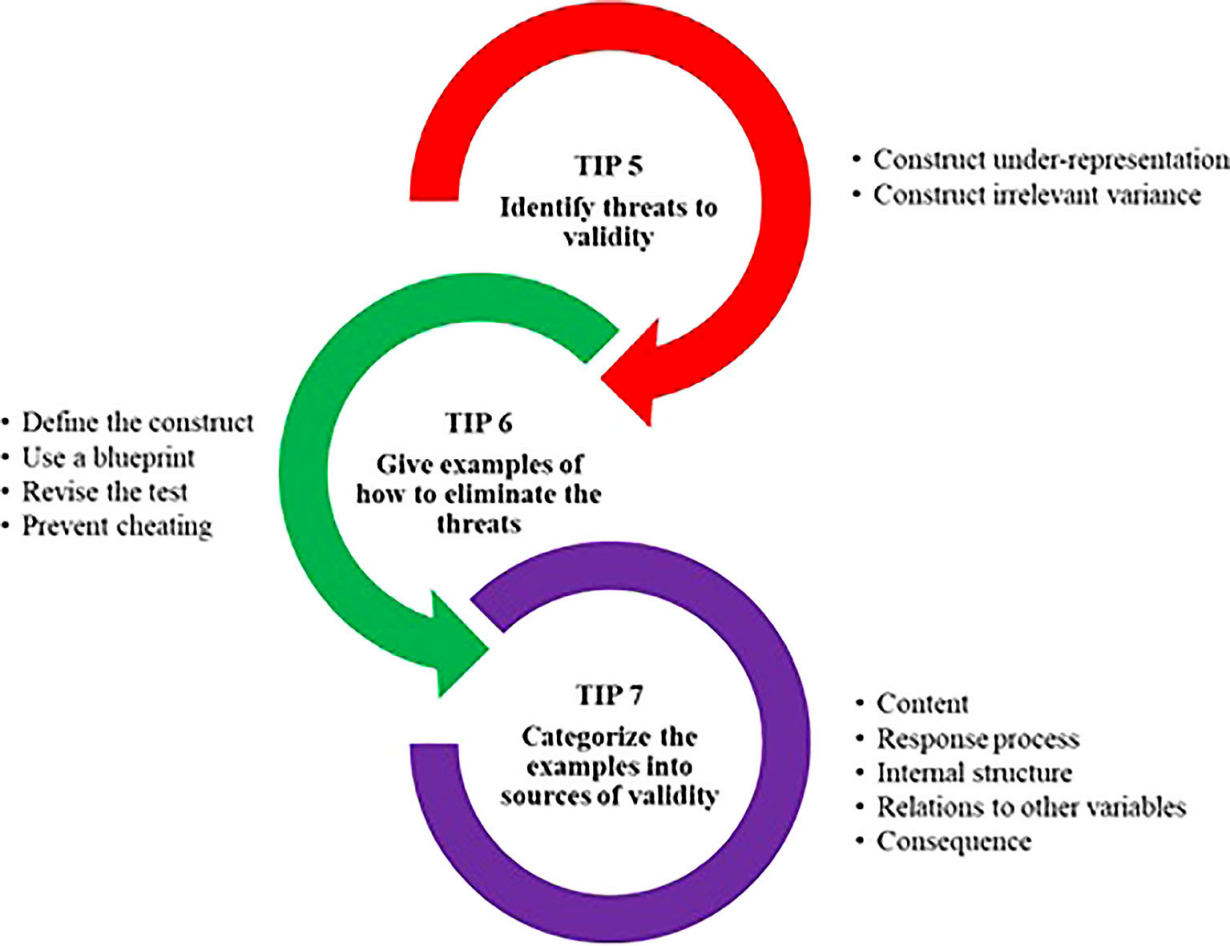
Summary of the process in Tips 5, 6, and 7.

This tip (Tip 6) will usually be interwoven with the previous tip and the following tips. The following diagram ( Figure 1) explains the process for Tips 5, 6, and 7.

## Tip 7. Ask the participants to provide examples of how to eliminate the threats to validity

Downing has provided a comprehensive guide describing the threats to validity, including examples of how to eliminate them ( Downing and Haladyna, 2004). These threats are divided into two categories: construct under-representation (CU) and construct-irrelevant variance (CIV). An example of the former category (CU) is the use of few test items, while the latter category includes any approach that leads to bias in the interpretation of test results (e.g., flawed items). Asking the participants to mention examples of actions taken to eliminate threats to validity will prompt them to think about providing evidence for those actions, which will include evidence of validity. This will pave the way for the introduction of the five sources of evidence of validity. Sources of validity evidence can be gathered according to the following categories: content, response process, internal structure, relationship to other variables, and consequences ( Downing, 2003). It is useful to conduct an exercise in which every possible piece of evidence is categorized into the five sources; such an exercise will maximize the participants’ engagement.

## Tip 8. End at the beginning: Explain the unitary concept of validity

Now that the participants are familiar with the history of the changes in the concept of validity, the notion of construct validity, validity threats, and the five sources of validity evidence, it is time to tie it all together and solidify the unitary concept of validity. By this point in the workshop, you will find that your job has become easy.

## Tip 9. Ask the participants to compare the tripartite concept of validity to the current concept of validity

By now, some of the participants will still be centered on the old concept of validity (that is based on the tripartite notion). ( Goodwin and Leech, 2003) provided a useful way to aid in the participants’ transition from the tripartite concept to the unitary concept by comparing the types of validity evidence between the old concept and the current concept. The purpose of this step is to ease the transition for the participants who are still in favor of the traditional concept.

## Tip 10. Address the misconceptions

The comparison between the tripartite concept and the current concept will very likely reveal the number of misconceptions that are still out there. Examples of misconceptions may include the use of the phrase *validity of a test* instead of *validity evidence that supports the interpretation* or the idea that *any amount (or type) of validity evidence is enough to build the case* rather than a focus on the importance of each piece of evidence in relation to the construct and the assessment tool. Furthermore, some may think that *validation is just a collection of evidence* rather than a whole process that includes the identification of the construct and an evaluation of the evidence. One of the false impressions is that validity is a dichotomy (it is either valid or not valid) as opposed to a continuum. Some estimate reliability and rely on it as the sole source of validity, while others think that *after establishing a validity argument for a test, the test is valid for any further/different uses.* Gasmalla and Tahir (2020) elaborated on these misconceptions.

## Tip 11. Ask the participants to validate the test scores from their own previously developed tests

At this point, it is time for the participants to apply what they have learned. Thus, it is necessary to ask them to bring any previous tests they have developed along with the test scores. They can work in groups. Their validation activity may raise some questions that were addressed previously, or it may reveal unnoticed misconceptions. As the instructor, you must be patient since the concept of validity is complex, ever-evolving, and the subject of continuous debate.

## Tip 12. Post-credit scene: Promote a culture of quality assurance

By the end of the workshop, participants may question the need to discuss such a complex concept. In this step, a culture of quality assurance should be promoted. Assuming the workshop has gone as planned, the participants will find themselves thinking about quality assurance at every step in their career in education. In fact, the promotion of quality assurance should be highlighted throughout the workshop.

## Conclusion

This work aims to provide guidance about educating novice medical teachers about the concept of validity argument with regard to assessments using a workshop. Critical reflection and a careful review of the literature were applied to develop these tips. The tips focused on the history of the evolution of the concept of validity, the notion of construct validity, validity threats, and the five sources of validity evidence; they also addressed the common misconceptions that accompany the application of validity. These tips were arranged in such a way to help the workshop participants understand the concept of validity.

## Take Home Messages

• The interpretation of the term validity has changed over time.
• Misconceptions and malpractices have been noted in the reporting of validity.
• Introducing the concept of validity requires a systematic approach. This approach includes a consideration of the historical evolution of the concept, a discussion on the threats to validity, an explanation of the unitary concept of validity, a comparison of the tripartite concept of validity with the current concept of validity, addressing misconceptions, and the promotion of a culture of quality assurance.
• The backbone of this approach involves conducting a TNA followed by an effective faculty development program.

## Notes On Contributors

**Hosam Eldeen Elsadig Gasmalla** is an assistant professor of human anatomy and histology in the faculty of Medicine, Al Neelain University and a researcher in the Education Development Center, Sudan International University. ORCID ID: https://orcid.org/0000-0003-2590-8587

**Majed Mohamed Saleh Wadi** is a senior lecturer of Medical Education, College of Medicine, University of Qassim, KSA. He is the Head of Medical Education Department. Head of the Assessment Committee. ORCID ID: https://orcid.org/0000-0002-8117-770X

**Mohamed Hassan Taha** is a visiting Assistant Professor of Medical Education at the College of Medicine and Medical Education Centre, University of Sharjah, UAE. He is the chair of the Curriculum Committee as well as the Faculty Development Committee. ORCID ID: https://orcid.org/0000-0003-0808-5590

## Figures and Tables

**Table (1):** Suggested timetable for the workshop for delivering the validity argument in assessment concept to novice medical teachers, facilitator’s version

Time/duration	Session/concept	Mode of delivery	Applied tip
8:00–8:30	Welcome, icebreakers		
8:30–8:45	Pertest		
8:45–10:00 AM	Validity in students’ assessments: What do we know about it? Historical background	Interactive session Debate	Tip #2 Explore the workshop participants’ prior knowledge Tip #3 Consider the historical evolution of the concept
10:00–10:30 AM	Coffee break		
	What is construct?	Interactive session Debate	Tip #4 Explain the concept of a construct
10:30–11:30 AM	Threats to validity and how to eliminate them	Group work Group presentation	Tip #5 Start backwards: Discuss the threats to validity Tip #6 Ask the workshop participants to define the impact of the threats on inference Tip #7 Start backwards: Discuss the threats to validity
11:30–12:30 PM	Validity concepts Validity as a unitary concept Previous and current concepts of validity: A comparison	Interactive session Debate	Tip #8 End at the beginning: Explain the unitary concept of validity Tip #9 Ask the participants to compare the tripartite concept of validity to the current concept of validity
12:30–13:00 PM	Coffee break		
13:00–14:00 PM	Addressing the misconceptions	Interactive session	Tip #10 Address the misconceptions
14:00–15:00 PM	Exercises	Group work Group presentation	Tip #11 Ask the participants to validate the test scores from their own previously developed tests Tip #12 Post-credit scene: Promote a culture of quality assurance
15:00–15:15 PM	Post-test		
15:15–15:30 PM	Wrap up/summary and take-home message		
