# Supplementary material for: Twelve tips for introducing the concept of validity argument in assessment to novice medical teachers in a workshop
Source: MedEdPublish (2016). 2021 Sep 21;10:74. Originally published 2021 Mar 25. [Version 2] doi: 10.15694/mep.2021.000074.2 (PMC10939636; doi:10.15694/mep.2021.000074.2)
Supplement: Peer review history

# Reviewer report: Reviewer response for version 2

**DOI:** 10.21956/mep.20213.r31297

**Published:** 2021-10-12

**Authors:** Julie Hunt

**Affiliations:** 1 Lincoln Memorial University

This review has been migrated. The reviewer awarded 4 stars out of 5 In this 12 tips article, the authors discuss how to structure and run a workshop teaching about current validity theory for educators. I appreciate the authors' method of introducing threats to validity and having educators work with those before grappling with validity frameworks, sources of evidence, etc. Overall this is an important topic for educators, as all of us make assessments of our students with varying implications for their progression, and these instructions would help educators or administrators to set up a workshop for faculty.

Reviewer Expertise:

NA

No decision status is available

# Reviewer report: Reviewer response for version 2

**DOI:** 10.21956/mep.20213.r31296

**Published:** 2021-09-22

**Authors:** Ken Masters

**Affiliations:** 1 Sultan Qaboos University

This review has been migrated. The reviewer awarded 4 stars out of 5 The authors have addressed most of my concerns about Version 1 of the article, and have produced an improved Version 2. I think that the article will be very useful for those who wish to run workshops on the topic. Well done.

Reviewer Expertise:

NA

No decision status is available

# Reviewer report: Reviewer response for version 1

**DOI:** 10.21956/mep.19018.r27297

**Published:** 2021-06-13

**Authors:** Ken Masters

**Affiliations:** 1 Sultan Qaboos University

This review has been migrated. The reviewer awarded 3 stars out of 5 An interesting 12-tips paper on introducing the concept of validity in assessment to novice medical teachers in a workshop.Overall, the paper is a nice blend of theoretical background and practical tips. Many important ideas are raised.There are, however, some gaps, and I would like to see these addressed:• Tip 1: Although restricting the number of people to 15 is generally a good idea in workshops, in most organisations, this is not feasible. The needs of the institution are so great, and running the workshop multiple times is usually not possible. So, although it would go against the advice of the authors, it would help if they could address the question of how to mitigate the problems if presenters were forced to run the workshop for 25-30 participants. (For many of these presenters, “It cannot be done” is not an option).• Similarly, the paper should give an idea of a timetable. While I understand that this may depend upon the results of the needs analysis, a rough guide (perhaps as an appendix) would be useful.• Figure 1: There is a typo (“elminat”). • Tip7 : It would be useful here to give a summary of Downing’s guide.• Tip 8 and elsewhere: As there are several crucial terms and definitions, I would like to see the paper summarise them, so that they could be used by presenters to be given to the participants. • Related to the point above: One of the roles of a 12-tips paper is that it can stand alone and assist the novice. The readers can trust that the authors have done all the background reading, and now present some form of summarised, off-the-shelf package that can be used. In many instances, this kind of practical assistance is offered in the paper, but there are gaps. Perhaps a way to test this is to give the paper to a person who knows little about the topic, ask them to use the paper to design such a workshop, and have them explain how things would be done. In this way, the gaps would become more evident and could be addressed.So, I would like to see Version 2 of the paper in which these issues are addressed.

Reviewer Expertise:

NA

No decision status is available

# Reviewer report: Reviewer response for version 1

**DOI:** 10.21956/mep.19018.r27296

**Published:** 2021-06-04

**Authors:** Barbara Jennings

**Affiliations:** 1 Norwich Medical School

This review has been migrated. The reviewer awarded 3 stars out of 5 Thank you to the authors for an interesting and thought-provoking article about the important topic of validity as a concept in assessment. The literature cited spans many decades & includes recent articles that consider problematic misconceptions - and throughout the article the idea of an evolving definition for validity (within medical education and assessment) is revisited.I think there are a few problems with the article in its current form however, and these could be considered by the authors for a revised version;1. The field is jargon-heavy and the introduction (and some tips in the main body) does (do) not have enough explanations in plain English for the general medical educator; or indeed, explicit descriptions of the concepts raised. For example, what are the precise threats to validity raised by Downing and others?2. The tips are presented with a notional workshop in mind. I am unclear about which participants the authors had in mind for such a workshop – could the authors be explicit about this or perhaps consider a more generalisable model. An assessment team may be expected to know a great deal about the topic but many medical educators and even module leads may not have any preconceived ideas or enough background knowledge to warrant working through the particular steps (though would need to know about the broad concept around validity and quality assurance). This takes me to concern number 3.3. Why focus on a history of the field at all in a workshop (tips 2/3)? Beginning with a unitary concept of validity from the start of any training seems the best approach to me – can we make progress if we constantly justify the evolution within a field? 4. A minor problem is typos and syntax issues to correct in abstract, main body and the figure.I think the article could be more successful if it presented “twelve tips about what medical educators should know about the concept of validity” – rather than a design for a very particular workshop that may not have wide-application?

Reviewer Expertise:

NA

No decision status is available
